# Analysis of the Cluster Prominence Feature for Detecting Calcifications in Mammograms

**DOI:** 10.1155/2018/2849567

**Published:** 2018-12-30

**Authors:** Alejandra Cruz-Bernal, Martha M. Flores-Barranco, Dora L. Almanza-Ojeda, Sergio Ledesma, Mario A. Ibarra-Manzano

**Affiliations:** ^1^Laboratorio de Procesamiento Digital de Señales, Departamento de Ingeniería Electrónica, DICIS, Universidad de Guanajuato, Carr. Salamanca-Valle de Santiago KM. 3.5 + 1.8 Km., Salamanca 36885, Mexico; ^2^Departamento de Ingeniería Robótica, Universidad Politécnica de Guanajuato, Av. Universidad Norte SN., Comunidad Juan Alonso, Cortazar 38496, Mexico; ^3^Cuerpo Académico de Telemática, DICIS, Universidad de Guanajuato, Carr. Salamanca-Valle de Santiago KM. 3.5 + 1.8 Km., Salamanca 36885, Mexico

## Abstract

In mammograms, a calcification is represented as small but brilliant white region of the digital image. Earlier detection of malignant calcifications in patients provides high expectation of surviving to this disease. Nevertheless, white regions are difficult to see by visual inspection because a mammogram is a gray-scale image of the breast. To help radiologists in detecting abnormal calcification, computer-inspection methods of mammograms have been proposed; however, it remains an open important issue. In this context, we propose a strategy for detecting calcifications in mammograms based on the analysis of the cluster prominence (*cp*) feature histogram. The highest frequencies of the *cp* histogram describe the calcifications on the mammography. Therefore, we obtain a function that models the behaviour of the *cp* histogram using the Vandermonde interpolation twice. The first interpolation yields a global representation, and the second models the highest frequencies of the histogram. A weak classifier is used for obtaining a final classification of the mammography, that is, with or without calcifications. Experimental results are compared with real DICOM images and their corresponding diagnosis provided by expert radiologists, showing that the *cp* feature is highly discriminative.

## 1. Introduction

Breast cancer is the top cancer that affects women both in developed and developing countries. Early detection of breast cancer increases treatment options and survival expectation [1]. Breast cancer statistics report that nearly 2 million of new cases were diagnosed in 2018; this represents about 12% of all new cancer cases and 25.3% of all cancers in women [2]. For an analysis of the efficiency in each stage of the cancer disease, the diagnosis, registration, and monitoring of diseases allow to validate the most appropriate treatments, including the optimization of costs [3]. The early detection for improving breast cancer outcome and survival remains an open issue.

The DICOM is the format used for registering a digitized mammographic image. Nowadays, mammography is a reliable method for breast cancer detection. In addition, several computer-aided detections (CAD systems) help mammogram processing to provide more accurate results [4]. A mammography is a low-energy radiography of the breast. The radiologist uses this method to localize morphological alterations and infers the presence or absence of anomalies, mainly small calcifications [5]. Breast calcifications are small spots of calcium salts in the breast tissue. The calcifications in the mammography appear as small white spots. There are two different types of calcifications, microcalcifications and macrocalcifications [6]. The macrocalcifications are large and coarse, mostly benign and associated with the age. The microcalcifications can be early signs of breast cancer, with or without a visible mass. This mass can be a benign tumor, a cyst, or cancer.

The detection of microcalcifications in mammograms is suboptimal because it depends on the radiologist's experience, criterion, fatigue, and visual capability. As a result, radiologists fail to detect breast cancers due to misinterpretation of the lesion and can lead to a greater number of false-positive cases. Another aspect is the accuracy with which the radiologist considers the medical importance of the calcification regions because sometimes these region's sizes can be misinterpreted in the mammograms [7]. Microcalcifications are bright spots whose size oscillates between 0.1 mm and 1 mm [8], and usually, they are not easy to see. Furthermore, only certain suspicious microcalcifications (<0.5 mm) are alarming, as it is verified in a comparative study between cancer size measurements and the results of pathology [9]. As a result, radiologists fail to detect breast cancers.

Several methods have been developed to assist the radiologist in the detection of calcifications using mammography images [10, 11] or computed aid detection (CAD systems). CAD systems have had significant technology advances yield to detect segmentation and classify microcalcification clusters at digital mammograms. Thus, CAD systems have been used clinically for more than two decades as “second lector” in the diagnosis carried out by the radiologists [9, 12]. The use of CAD systems is popular due to the high sensitivity detection averaging up to 90% [13]. On the other hand, the images obtained during the acquisition of a mammography are low contrast, making the processing a challenging task. Some methods are proposed to segment several types of microcalcifications using texture features. Kim and Park [14] compared the surrounding region-dependence method (SRDM) to the other conventional texture-analysis methods with respect to detection of clustered microcalcifications in digitized mammograms. The performance results of the classification are usually evaluated using receiver operating curve (ROC) curve that describes the discrimination capacity of the approach [15]. Yadollahpour and Hamed in [16] presents a review of various methods considering texture analysis for mass and microcalcification detection in mammography used for early breast cancer detection. Jalalian et al. in [17] obtains statistical texture feature based on the co-ocurrence matrix from a segmented volume of interest. The classification stage uses a multilayer perceptron neural network achieving high accuracy results.

Both applications Hough transform and threshold-based method were considered by Fanzinni et al. [18] as strong techniques that yield to group single microcalcifications, as “success events,” into clusters using a set of expert codified rules. From this, it is possible to obtain the regions that contain the lesions of interest. High-frequency filters were used by Lauria et al. [19] as preprocessing filters to carry out the segmentation of possible suspicious areas at mammography. The microcalcification analysis yield to find and classify regions of interest (ROIs) by means of two neural networks: the first is a feedforward neural network, while the second neural network uses the principal component method to end the process of classification. Samala et al. in [20] presents a study corresponding to the advantages and challenges for detection of microcalcifications in digital mammograms and digital breast tomosynthesis from a CAD systems perspective.

Basic concepts of mathematics define an inflection point as a point on the curve at which the sign of the curvature (i.e., the concavity) changes. In this approach, such changes on the curvature represent the fluctuations/variations of agglomerations in the image with respect to the specific level of the *cp* texture feature. In this paper, the analysis of the cluster prominence *cp* texture feature on mammography images is carried out to detect breast calcifications that may indicate cancer. The modelling of the last part of the *cp* histogram reveals the presence of microcalcifications with respect to another common mass on the breast tissue. The final classification of the mammography is performed using a weak classifier. The document is organized as follows: the next section describes the proposed strategy for detecting calcifications. Also, it explains the *cp* feature computation, the global and local interpolation, and the classification of the *cp* histogram. The experimental results and conclusions are, respectively, provided in the last part of the document.

## 2. Materials and Methods

**Figure 1:**
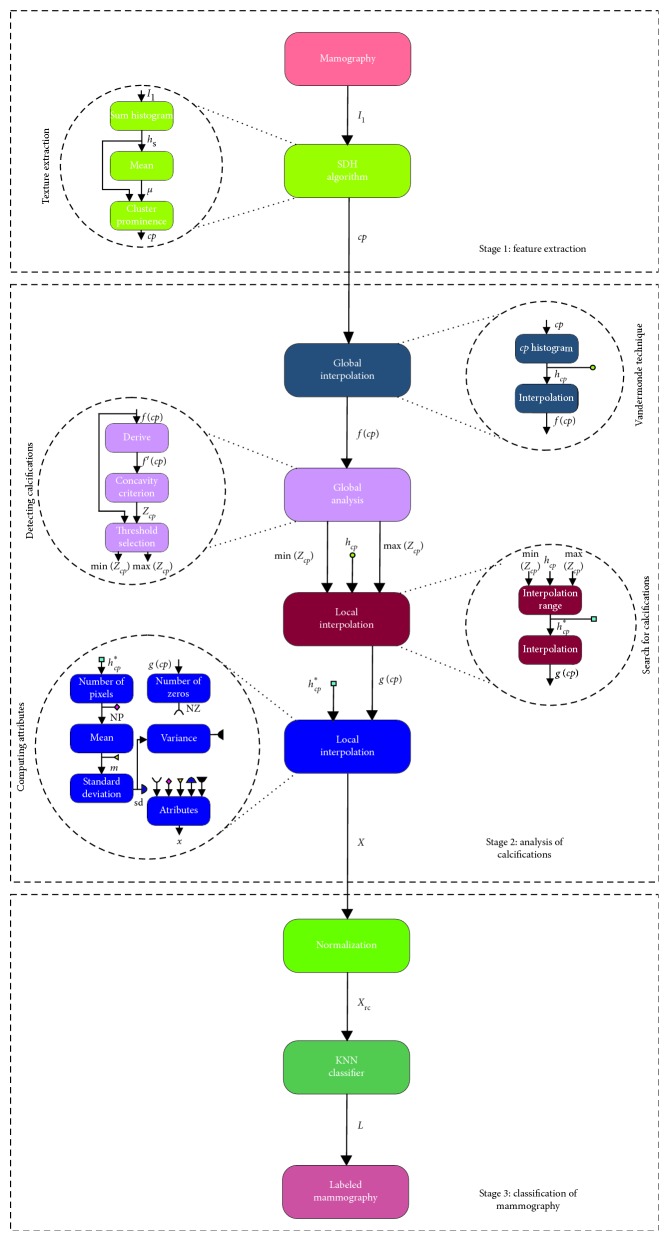
Block diagram of methodology to detect calcifications in mammograms.

The analysis of the cluster prominence (*cp*) texture feature is proposed to accurately detect calcifications in digital mammography. The proposed approach described here uses the mammography dataset collected at the General Hospital of Irapuato, Guanajuato. The dataset consists of 74 images: 22 diagnosed with calcifications and 52 diagnosed as normal tissue, in accordance with the classification system for breast mammogram, BI-RADS. An overview of our proposed approach is depicted in Figure 1, presenting mainly three stages: (1) feature extraction, (2) analysis of calcifications, and (3) classification of the mammography. First, the *cp* texture feature is computed from a mammogram using the sum and difference histograms (SDH) technique [21].

This feature is a measure of asymmetry for which a high value indicates large changes in the gray-scale levels of the image [22]. In other words, *cp* is a measure of how uniform is the gray-scale level distribution. In accordance with this, it is proposed that high *cp* values represent calcifications that can be more deeply analysed for validating the detection of calcifications.

The *cp* feature histogram is analysed using the Vandermonde technique. A global interpolation function *f(cp)* that best describes the *cp* feature histogram is obtained. This function is used to analyse the range of the histogram with high possibilities of finding calcifications, by means of the *n* inflection points in *f(cp)*, here referred as *Z*_*cp*_. Each inflection point represents different and noticeable agglomeration zones of the mammography with a high level of *cp* texture feature. However, a deeper analysis must be performed to search and validate the presence of calcifications on one specific zone of the high frequency. To accomplish this, the *g(cp)* function is obtained from a local interpolation on the high frequency range of the *cp* histogram. Using a specific range of the *g(cp)* function, the attributes *X* vector is computed.

The stage of mammograms classification receives the normalized version of statistical attributes (including the number of zeros) of the *X* vector and then classified using the K-nearest neighbour. The classification results refer the mammogram as “*with calcifications*” or “*without calcifications*.” In the following, these stages will be explained on detail.

### 2.1. Texture Feature Extraction Using SDH Algorithm

The sum and difference histogram (SDH) technique calculates histograms that collect the results of addition and subtraction of the gray-scale levels on a whole digital image [18]. The SDH requires basic arithmetic operations and less memory storage in comparison with other texture techniques. Furthermore, it stores important information about the image content.

Considering the mammogram image as a rectangular matrix of size *K* × *L*, the sum image *I*_S_ is obtained by summing each pixel and their surrounded pixels are separated by a set of *M* relative displacements. The gray level at each pixel is quantified to *N*_g_ levels; therefore, the range of the *I*_S_ image is [0, 2(*N*_g_−1)]. From the *I*_S_ image, the sum histogram (*h*_S_) is calculated for a rectangular window of *N* elements (*N* = width × height, 3 × 3) storing the cardinality at each coordinate (*x*, *y*) in the window with an intensity value *i*. Finally, the normalized sum histogram $\hat{P_{S}}\left(i\right)$ is given by the following equation:

(1) $$\begin{array}{c} \hat{P_{S}}\left(i\right)=\frac{h_{\text{S}}\left(i\right)}{N}. \end{array}$$

The *cp* feature is given by the following equation:

(2) $$\begin{array}{c} cp=\underset{i}{\sum }{\left(i-2\mu \right)}^{4}\cdot \hat{P_{S}}\left(i\right). \end{array}$$

As mentioned above, this feature is used to seek for calcifications in mammograms, as calcifications and agglomerations are related with high values in the *cp* texture feature.

The size of the region used for computing the *cp* feature is *D* *=* 3 × 3 because the calcifications are more visible using this size. The displacement was established only in the horizontal direction, although more directions were evaluated, i.e., 45°, 90°, and 135° without noticeable changes. The value was set at “1” because as mentioned above, the sizes of the microcalcifications are <0.5 mm [9]. This minimal resolution of “1” pixel allows to detect the microcalcifications.

### 2.2. Analysis of the *cp* Feature Histogram

In this section, it explains the theoretical context of the Vandermonde technique, and the global and local interpolations are performed for modelling the *h*_*cp*_ behaviour. The histogram of the *cp* feature (here denoted as *h*_*cp*_) might be numerically modelled by a polynomial function *f(cp)* that globally describes the histogram behaviour. Such *f(cp)* function is obtained using the Vandermonde interpolation technique. After that, a local interpolation is performed (using the same interpolator) but only for high values of *cp* features.

#### 2.2.1. Vandermonde Technique for Global Interpolation

The basic procedure to determine the coefficients *a*_0_, *a*_1_, …  , *a*_*n*_ of the polynomial function

(3) $$\begin{array}{c} P_{n}\left(x\right)=a_{0}+a_{1}x+a_{1}x^{2}+,\;\;\ldots \;\;,+{\;a}_{n}x^{n} \end{array}$$

consists in interpolating the *m*+1 points (*x*_0_, *y*_0_), (*x*_1_, *y*_1_),…, (*x*_*m*_, *y*_*m*_) yielding a linear system of equations

(4) $$\begin{array}{c} P_{n}\left(x_{0}\right)=y_{0\;\;}⟶a_{0}+a_{1}x_{0}+a_{2}x_{0}^{2}+\ldots +a_{n}x_{0}^{n}=y_{0},\\ P_{n}\left(x_{1}\right)=y_{1\;\;}⟶a_{0}+a_{1}x_{1}+a_{2}x_{1}^{2}+\ldots +a_{n}x_{1}^{n}=y_{1},\\ \vdots \;⟶\;\vdots \;\\ P_{n}\left(x_{m}\right)=y_{m\;\;}⟶a_{0}+a_{1}x_{m}+a_{2}x_{m}^{2}+\ldots +a_{n}x_{m}^{n}=y_{m}, \end{array}$$

or in matrix form $V\bar{a}=\bar{y}$.

(5) $$\begin{array}{c} \left(\begin{array}{ccccc} 1 & x_{0} & x_{0}^{2} & \cdots & x_{0}^{n}\\ 1 & x_{1} & x_{1}^{2} & \cdots & x_{1}^{n}\\ \vdots & \vdots & \vdots & \cdots & \vdots \\ 1 & x_{m} & x_{m}^{2} & \cdots & x_{m}^{n} \end{array}\right)\left(\begin{array}{c} a_{0}\\ a_{1}\\ \vdots \\ a_{n} \end{array}\right)=\left(\begin{array}{c} y_{0}\\ y_{1}\\ \vdots \\ y_{m} \end{array}\right). \end{array}$$

The matrix *V* of this linear system is called *Vandermonde matrix*. As this matrix is nonsingular, the system $V\bar{a}=\bar{y}$ could be solved to obtain the coefficients $\bar{a}=\left(a_{0},\;\;a_{1},\;\;\ldots ,a_{n}\right)$. In this work, the *f(cp)* function obtained from the Vandermonde interpolation is a polynomial function of order 13. This function is used to obtain the range of the histogram with high possibilities of finding calcifications. To accomplish this, the first derivative of *f(cp)* is computed, while its *n* inflection points *Z*_*cp*_:

(6) $$\begin{array}{c} Z_{cp}=\left\{cp_{i}\;|\;f^{\prime }\left(cp\right)=0,\;i=1,2,\ldots ,n\right\}. \end{array}$$

**Figure 2:**
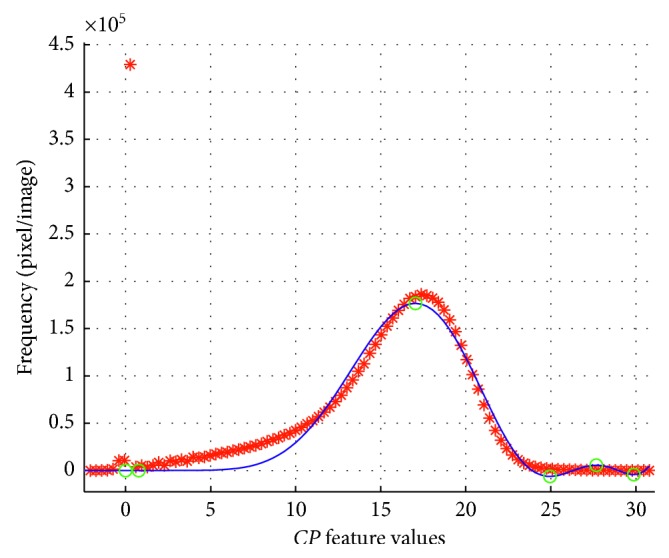
Global interpolation of *h*_*cp*_. The asterisks ∗ depict the discrete values of *h*_*cp*_, the continuous blue line is the interpolated polynomial function *f(cp)*, and circles illustrate the inflection points.

Figure 2 depicts in asterisks (*∗*) the interpolated function *f(cp)* and in circles (*o*) the corresponding inflection points of the function. Note that *f(cp)* follows the shape of *h*_*cp*_ (continuous line), except at the highest values of the *cp* feature that shows minimal and maximal local frequency values. Therefore, a second interpolation must be performed to fit the *h*_*cp*_ behaviour in the last part of the function.

### 2.3. Global Analysis: Detecting Calcifications

**Figure 3:**
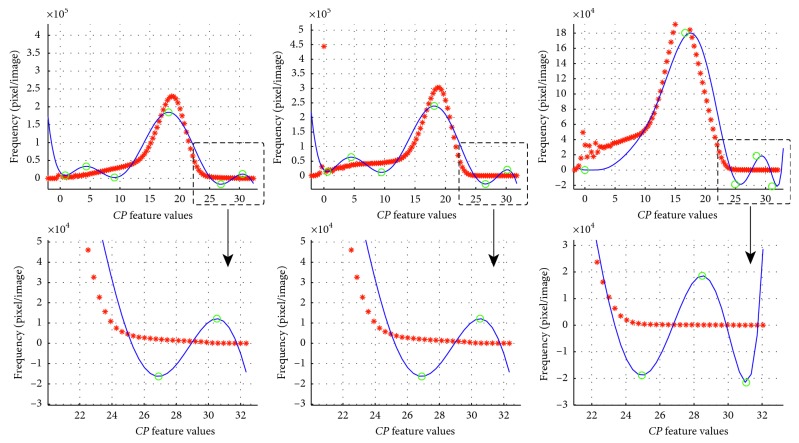
Behaviour of the high frequency part of different *f(cp)* functions. First row: the histograms *h*_*cp*_ (red asterisks) and polynomial interpolation *f(cp)* (blue continuous line) computed from different mammograms diagnosed with calcifications. Second row: a zoom inside of the last part of the graph. The circles represent *Z*_*cp*_.

Note in Figure 3 that to calculate the minimal and maximal threshold *th*_min_ and *th*_max_, it is necessary to analyse two different behaviours on the last part of the *f(cp)* function: (1) when the last inflection point in *Z*_*cp*_ is maximal and the function decreases; (2) when the last inflection point in *Z*_*cp*_ is minimal and the function increases. The behaviour 1 is illustrated by the first two graphs of Figure 3, and the last graph illustrates the second behaviour case.

In this work, we propose a concavity criterion to choose the threshold values. The concavity criterion is established as follows: the searching zone for calcifications is ranged from the penultimate inflection point to the last inflection point if the last inflection point is maximal, otherwise is ranged from the antepenultimate inflection point if the last inflection point is minimal:

(7) $$\begin{array}{c} {\text{th}}_{\min}=\left\{\begin{array}{c} cp_{\left(n-1\right)},\;\text{if}\;\;f\left(cp_{n}\right)>f\left(cp_{n-1}\right),\\ cp_{\left(n-2\right)},\;\text{otherwise}. \end{array}\right. \end{array}$$

In both cases illustrated in Figure 3, the maximal th_max_ is the last inflection point. The range established by th_min_ and th_max_ is used for obtaining a second interpolation function of *h*_*cp*_. This function will be referred as *g(cp)*, and it is interpreted as the result of a local interpolation.

### 2.4. Local Interpolation: Search for Calcifications

The part of the histogram *h*_*cp*_ located in the ranges *th*_min_ and *th*_max_ is referred as *h*_*cp*_^*∗*^. In this local interpolation, the function *g(cp)* fits the *h*_*cp*_^*∗*^ curve values more accurately and is used to calculate the new inflection points of *Z*_*cp*_.

**Figure 4:**
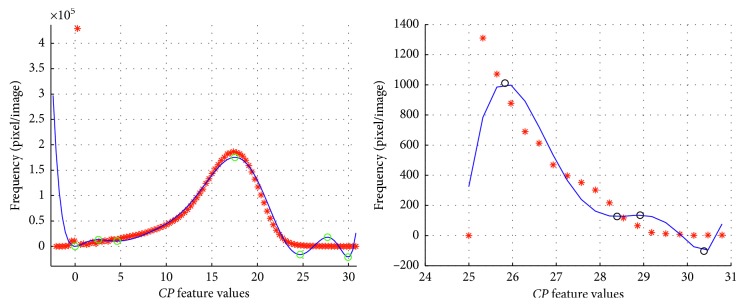
Comparing global and local interpolation: (a) global interpolation of *h*_*cp*_; (b) local interpolation of *h*_*cp*_^*∗*^, where new inflection points were found and the variability of *h*_*cp*_ is noticeable.

In Figure 4, the global and local interpolation results are compared. Figures 4(a) and 4(b) show, respectively, the global and local interpolation function *f(cp)* and *g(cp)*. Note that after the local interpolation process, the function *g(cp)* (the blue continuous line in Figure 4(b)) fits better the *h*_*cp*_^*∗*^ values (red asterisks) than in the last part of the global interpolation function of Figure 4(a). Furthermore, the new *g(cp)* function includes additional inflection points in *Z*_*cp*_ (black circles in Figure 4(b)), which better describes the high frequency changes. This variability during second interpolation allows to suppose the presence of calcifications in the mammogram; however, the result will be delivered by the classifier.

#### 2.4.1. Local Analysis for Computing Attributes

The *g(cp)* function is used for computing some texture attributes (*X* vector) on the zone of the DICOM image in which the calcifications could be found. The attributes computed are (1) the number of zeros *Z*_*cp*_ and (2) the number of pixels contained in *h*_*cp*_^*∗*^. From the second attribute, it is possible to compute three statistical values, like mean, standard deviation, and variance yielding 5 attributes in total. Such attributes are discriminant enough to be used in the classification process of normal and malign tissue.

### 2.5. Classification of Mammography

The five attributes are normalized by the centered-reduced data technique and used as the input to a KNN classifier based on Euclidean distance. The KNN classifier ranks third as the most used classifier in the last 20 years in the mammography analysis [23], and in our case, due to the model proposed here, this classifier is the most appropriate. The Euclidean distance is sufficiently discriminant because the attributes are not linear, allowing a greater separation among classes. To unbalance the single data-classes, *K* must be chosen as an odd number, in our case *K* *=* 3. The classifier was trained and tested using leave-one-out cross validation (LOOCV). This validation technique ensures a very low error; additionally, it is typically used for small databases, providing a maximum adjustment of the training set and independent test set [23, 24].

## 3. Results and Discussion

**Figure 5:**
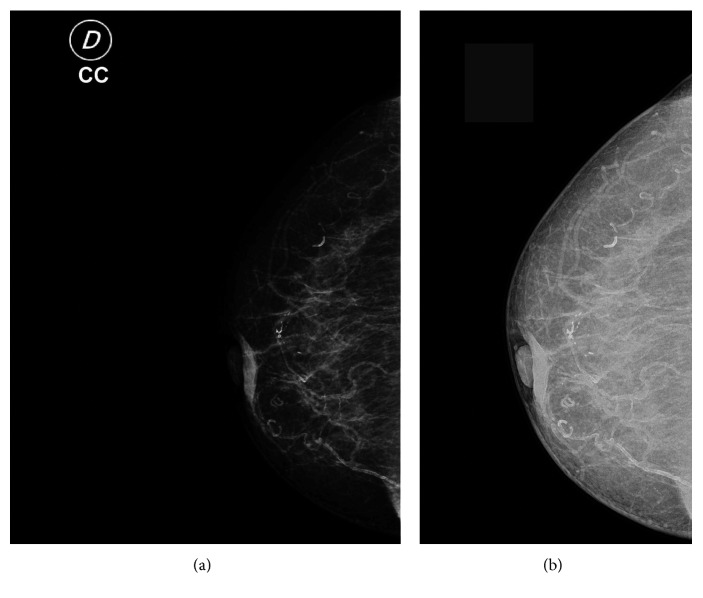
(a) Mammogram in the DICOM format, (b) *cp* feature of the mammogram (a).

As was described above, the mammography dataset consists of 74 images: 22 diagnosed with calcifications and 52 diagnosed as normal tissue, in accordance with the classification system for breast mammogram, BI-RADS. The mammograms are images stored as DICOM 3.0 format with a size of 4784 × 3517 pixels, as shown in Figure 5(a).  Figure 5(b) shows the *cp* attribute of the mammogram, used in this work for modelling sharp changes in the intensity.

**Figure 6:**
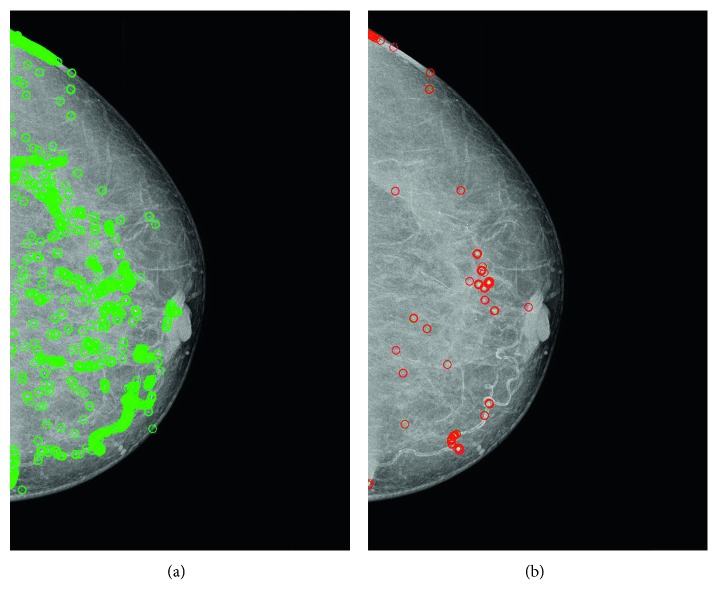
Calcification detection results: (a) calcifications and normal tissue detected with the global interpolation; (b) the local interpolation results only highlight the calcifications.

The results obtained from the global interpolation of the *h*_*cp*_ histogram are illustrated on Figure 6(a). Note that, this function highlights mass, conducts, calcifications, and healthy tissue at the same time. As we have mentioned above, a second interpolation technique is required for the high frequency zone of the *cp* histogram, yielding the *g(cp)* function.  Figure 6(b) illustrates the calcification detected by the *g(cp)* function. However, this second analysis still needs to validate which of these spots found belong to abnormal tissue growth or mass, that is, if these spots are calcifications or not. To accomplish this, five attributes are computed and analysed from the *g(cp)* function, and they are used as input to the KNN classifier.

**Figure 7:**
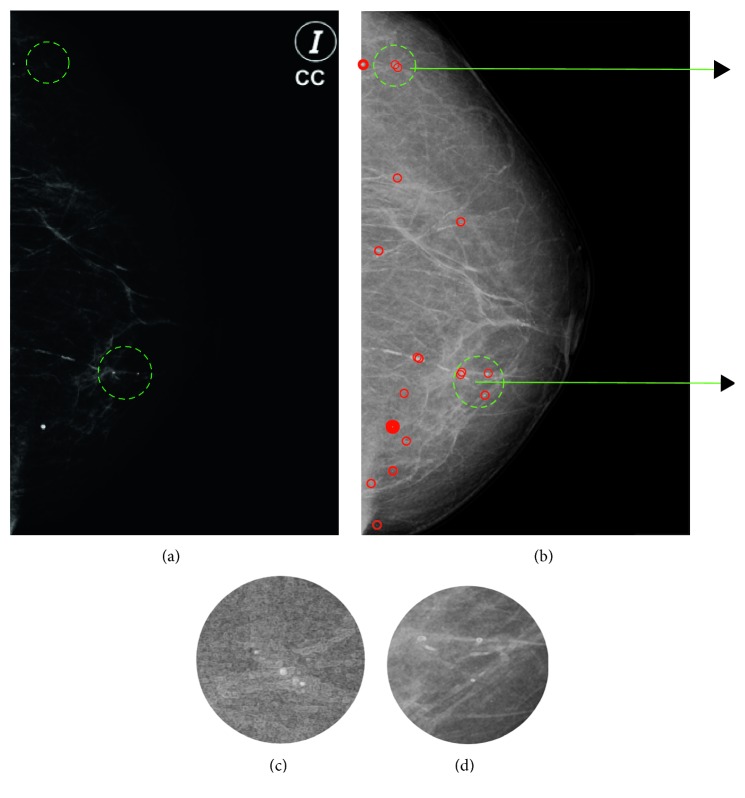
Classification results: (a) mammography in the DICOM format, the circles highlight the zones with calcifications found by an expert; (b) the *cp* attribute with the calcifications found by the proposed method; (c) and (d) zoom into the microcalcifications detected.

Figure 7(a) illustrates the classification results obtained by an expert, and Figure 7(b) illustrates the results obtained using our method. Our method detects exactly the two calcifications indicated by the expert in Figure 7(a). The *cp* feature detects big changes of intensity on the mammography; therefore, it is possible to detect more calcification on Figure 7(b) than those detected by the human eye. Figures 7(c) and 7(d) show the clusters of the microcalcifications found.

**Figure 8:**
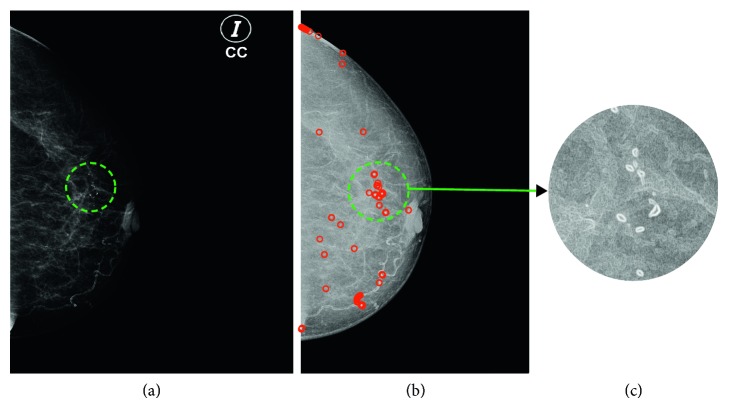
Classification results: (a) mammography in the DICOM format, circles highlight the zones with calcifications found by an expert; (b) *cp* attribute with the calcifications found by the proposed method; (c) zoom into the microcalcifications detected.

A second result is illustrated in Figure 8. Here, the calcifications visually found by an expert are also entirely detected by the proposed method.

Figures 7 and 8 illustrate qualitatively the correct performance of the proposed approach with respect to mammograms previously diagnosed with calcifications. Following subsection will show the quantitative results and a comparative table to point out the relevance of our proposed strategy.

### 3.1. Metrics of Performance Analysis

The quantitative results obtained by the experimental tests are summarized in the confusion matrix shown in Table 1. The worst case of a classifier is the false-negative (FN) score, that refers to the cases in which the classifier does not detect the calcification, and it exists. From Table 1, two false negatives (FNs) are obtained from the experimental tests. On the other hand, four false positive (FP) have been obtained from the experimental tests, and this result indicates the presence of calcifications that does not exist, in accordance with the diagnosis of the medical expert.

As reported in the confusion matrix, the specificity obtained from the experimental results is 0.9230 for normal tissue detection and 0.9090 of sensitivity for calcifications detection. Such rate of classification is usually compared with other strategies proposed in the literature. The worst case during the classification stage is due to the presence of false-negative values. In this case, two of the FN cases were analysed, and in both cases, the mammography shows low brightness.

Now, the choice of the polynomial function order is essential to warranty the best accuracy results. Thus, Table 2 shows the performance evaluation of our approach measured with different metrics including accuracy, precision, specificity, sensitivity, and percentage of false alarms (FA), for different orders of the polynomial function. The best accuracy value is 0.9189, for the 13 and 14 order of the function; however, the 13 order was chosen for simplicity. For this order, the obtained precision value is 0.8333 which is not as high as we would expect due to the false positives. This means that the most common error of our strategy occurs when a calcification is detected, but it does not exist. Such results are also validated by the specificity and the sensitivity values: 0.9230 and 0.9090, respectively. The percent of positive and negative incorrect detections (false alarms, FA) is 0.0769, and a low percentage of false negative is the most expected result.

A comparative analysis among different methods found in the state-of-the-art is shown in Table 3. The first column presents the techniques used by different authors including our proposed strategy, and the second column shows the dataset used for the experimental results. The third column indicates how the image processing is performed, which can be manual, semiautomated, and automated. The last column shows the achieved accuracy for each method.

Furthermore, in these methods, the algorithms processing is automated allowing the use of such strategies for real applications. However, we want to point out that in the case of methods that compute texture features, such as [20] and our approach, the best performance is shown by our strategy. Additionally, among these three methods, our approach is the only method that process images as automated.

Table 3 shows a comparative analysis between different related works in the state of the art. The first column lists the related methods, and all these methods use texture or appearance features for detecting calcifications and different classification methods. The dataset used for experimental tests is shown in the second column, and the third column indicates the execution mode of the method, that is automatic (A) and semiautomatic (S). Automatic refers that no intervention is necessary from the user; on the other hand, the semiautomatic requires a minimum intervention from the user. Note that, only the methods proposed in [26, 27] are semiautomatic.

The fourth column shows the accuracy value that allows us to compare the performance between each of the methods. In particular, our proposed approach and the works proposed in [15, 18, 19, 25] show an accuracy higher than 0.9, indicating a high-performance evaluation. Although our proposal does not present the best accuracy, it is positioned in the middle of all the high-performed approaches, being a good and competitive solution in general. Note that, the approach with the best accuracy, presented in [15], only differs by 0.0178 with respect to ours, and this difference can be solved using a dataset with a greater number of samples. The fifth column shows the sensitivity, which evaluates the performance only when a calcification is detected. For most of the compared works, this measure is superior to 0.9, in particular the work proposed in [25] shows the highest sensitivity, and our proposed method is only 0.06 below it. Finally, the sixth column shows the false positives per image (FPi), which allows to measure the errors in calcifications detection, that is, when the normal tissue is detected as a calcification. For this measure, all listed references are less than 6, indicating a good FPi performance.

## 4. Conclusions

A method for detecting calcifications based on cluster prominence *cp* feature analysis on mammograms is proposed in this work. A deep analysis about the cluster prominence feature throws that is highly discriminative and allows the modelling of the calcifications in comparison with other attributes. The classification error obtained is low. Among this misclassification, the most common error of our strategy is the false detection, that is the false positives, which finally is less critical than the false-negatives results. A performance comparison demonstrates that our proposed strategy has better performance than similar works. This methodology is proposed as a tool for helping the radiologist during diagnosis. A web application is under construction for providing a more flexible support to the final user. Future works include further experimental tests of this approach using INbreast dataset and a new characterization of the *cp* feature for improving time performance results.

## Figures and Tables

**Table 1:** Confusion matrix of the *g(cp)* function for *K* = 3 and using the Euclidean distance.

Desired/estimated	Normal tissue	Calcification	Specificity
Normal tissue	48 (TN)	4 (FP)	0.9230
Calcification	2 (FN)	20 (TP)	0.9090

**Table 2:** Metrics performance results of the proposed method for different orders of the polynomial function.

Order	Accuracy	Precision	Specificity	Sensitivity	% FA
9	0.8648	0.8333	0.9423	0.6818	0.0576
10	0.9054	0.8571	0.9423	0.8181	0.0576
11	0.8513	0.8333	0.9411	0.6818	0.0588
12	0.8378	0.9166	0.9807	0.5000	0.0192
13	0.9189	0.8333	0.9230	0.9090	0.0769
14	0.9189	0.8333	0.9230	0.9090	0.0769
15	0.9054	0.8226	0.9230	0.8636	0.0769

**Table 3:** Comparison between our methodology and state-of-the-art methods.

Compared methods	Dataset	Set-up	Accuracy	Sensitivity	FPi
Proposed strategy	Hosp. Irapuato	A	0.9189	0.9090	5.4054
PSOWNN [15]	Clinical	A	0.9367	0.9416	1.9006
DEOWNN [25]	MIAS	A	0.9353	0.9690	5.9754
Hough transform [18]	BCDR	A	0.9326	0.9178	3.9999
CALMA-ANN [19]	CALMA	A	0.9200	0.9200	4.9627
CAD-PPJ [20]	U of M.	A	0.8914	0.8500	1.7100
Texture feature + SLDA [26]	DDSM	S	0.8700	0.9333	10.000
Level set [27]	U. of M.	S	0.8500 ± 0.0200	—	—
Active contour [27]	U. of M.	S	0.8700 ± 0.0200	—	—

## Data Availability

Mammograms database and their corresponding medical diagnosis were provided by the Hospital Regional of Irapuato based on a privacy agreement with the Digital Signal Processing Laboratory of the University of Guanajuato. The agreement avoids publicly sharing or distributing any kind of data or mammography provided by the hospital.

## References

[1] World Health Organization (WHO) (May 2018). Breast cancer: prevention and control. http://www.who.int/topics/cancer/breastcancer/es/index1.html.

[2] World Cancer Research Fund International (October 2018). Global cancer observatory. http://gco.iarc.fr/today/.

[3] Katrakazas P., Costarides V., Tarousi M. Business process modelling for a Greek hospital’s medical equipment data center.

[4] Suzuki K. (2017). Overview of deep learning in medical imaging. *Radiological Physics and Technology*.

[5] Yoshino Y., Miyajima T., Lu H. (2017). Automatic classification of lung nodules on MDCT images with the temporal subtraction technique. *International Journal of Computer Assisted Radiology and Surgery*.

[6] Wang J., Nishikawa R. M., Yang Y. (2017). Quantitative comparison of clustered microcalcifications in for-presentation and for-processing mammograms in full-field digital mammography. *Medical Physics*.

[7] Lopez-Aligue F. J., Poveda-Pierola A., Acevedo-Sotoca I. Detection of micro-calcifications in digital mammograms.

[8] Jalalian A., Mashohor S. B. T., Mahmud H. R., Saripan M. I. B., Ramli A. R. B., Karasfi B. (2013). Computer-aided detection/diagnosis of breast cancer in mammography and ultrasound: a review. *Clinical Imaging*.

[9] Cheung Y.-C., Tsai H.-P., Lo Y.-F., Ueng S.-H., Huang P.-C., Chen S.-C. (2016). Clinical utility of dual-energy contrast-enhanced spectral mammography for breast microcalcifications without associated mass: a preliminary analysis. *European Radiology*.

[10] Arevalo J., Gonzalez F. A., Ramos-Pollán R. (2016). Representation learning for mammography mass lesion classification with convolutional neural networks. *Computer Methods and Programs in Biomedicine*.

[11] Hari V., Jagathy R., Gopikakumari R. Enhancement of calcifications in mammograms using Volterra series based quadratic filter.

[12] Wei L., Yang Y., Nishikawa R. (2017). Improved cancer detection in automated breast ultrasound by radiologists using computer aided detection. *European Journal of Radiology*.

[13] Cheng H. D., Shi X. J., Min R., Hu L. M., Cai X. P., Du H. N. (2006). Approaches for automated detection and classification of masses in mammograms. *Pattern Recognition*.

[14] Kim J. K., Park H. (1999). Statistical textural features for detection of micro-calcifications in digitized mammograms. *IEEE Transactions on Medical Imaging*.

[15] Dheeba J., Albert Singh N., Tamil Selvi S. (2014). Computer-aided detection of breast cancer on mammograms: a swarm intelligence optimized wavelet neural network approach. *Journal of Biomedical Informatics*.

[16] Yadollahpour A., Hamed S. (2015). Early breast cancer detection using mammogram images: a review of image processing techniques. *Biosciences Biotechnology Research Asia*.

[17] Jalalian A., Mashohor S., Mahmud R., Karasfi B., Iqbal Saripan M., Ramli A. R. (2017). Computer-assisted diagnosis system for breast cancer in computed tomography laser mammography (CTLM). *Journal of Digital Imaging*.

[18] Fanizzi A., Basile T. M. A., Losurdo L. Hough transform for clustered microcalcifications detection in full-field digital mammograms.

[19] Lauria A., Palmiero R., Forni G. (2004). The CALMA system: an artificial neural network method for detecting masses and microcalcifications in digitized mammograms. *Nuclear Instruments and Methods in Physics Research Section A: Accelerators, Spectrometers, Detectors and Associated Equipment*.

[20] Samala R. K., Chan H.-P., Hadjiiski L. M., Helvie M. A. (2016). Analysis of computer-aided detection techniques and signal characteristics for clustered microcalcifications on digital mammography and digital breast tomosynthesis. *Physics in Medicine and Biology*.

[21] Ibarra-Manzano M. A., Devy M., Boizard J. L. Real-time classification based on color and texture attributes on an FPGA-based architecture.

[22] Curran W. J., Liu T. (2012). Ultrasound glcm texture analysis of radiation-induced parotid-gland injury in head-and-neck cancer radiotherapy: an in vivo study of late toxicity. *Medical physics*.

[23] Pedro R. W. D., Machado-Lima A., Nunes F. L. S. (2019). Is mass classification in mammograms a solved problem? - a critical review over the last 20 years. *Expert Systems with Applications*.

[24] Jain A. K., Duin P. W., Mao J. J. (2000). Statistical pattern recognition: a review. *IEEE Transactions on Pattern Analysis and Machine Intelligence*.

[25] Dheeba J., Tamil Selvi S. (2011). An improved decision support system for detection of lesions in mammograms using differential evolution optimized wavelet neural network. *Journal of Medical Systems*.

[26] Ball J. E., Bruce L. M. Digital mammographic computer aided diagnosis (cad) using adaptive level set segmentation.

[27] Shi J., Sahiner B., Chan H.-P. (2007). Characterization of mammographic masses based on level set segmentation with new image features and patient information. *Medical Physics*.

